# Draft genome sequences of *Schizosaccharomyces pombe* strain I-540 isolated from grape must

**DOI:** 10.1128/mra.01135-24

**Published:** 2024-12-27

**Authors:** Egor A. Vasyagin, Alexey V. Beletsky, Elena V. Ivanova, Maxim Yu. Shalamitskiy, Andrey V. Mardanov, Nikolai V. Ravin

**Affiliations:** 1 Institute of Bioengineering, Research Center of Biotechnology of the Russian Academy of Sciences, Moscow, Russia; 2 Research Institute of Viticulture and Winemaking “Magarach” of the Russian Academy of Sciences, Yalta, Russia; University of California Riverside, Riverside, California, USA

**Keywords:** genome, *Schizosaccharomyces pombe*, maloalcoholic fermentation

## Abstract

*Schizosaccharomyces pombe* is a non-Saccharomyces yeast that is widely used in winemaking due to its ability to ferment malic acid, thus improving organoleptic properties of wine. We report the draft genome sequence of *S. pombe* strain I-540, isolated from grape must in Russia.

## ANNOUNCEMENT

Currently, there is a growing interest in the utilization of non-Saccharomyces yeasts to enhance oenological characteristics in wine production. Among them, *Schizosaccharomyces pombe*, owing to the unique features of its metabolism, has been explored for potential applications in winemaking (1). One of the key advantages of *S. pombe* is its ability to carry out maloalcoholic fermentation (MAF), a process in which a set of enzymes transform malic acid into CO_2_ and ethanol (2). Consequently, these yeasts can be employed for deacidification of wine, degrading up to 100% of malic acid (3). Furthermore, *S. pombe* strains contribute to the reduction of the formation of biogenic amines and ethyl carbamate, a probable human carcinogen (4), thereby enhancing the safety of wine. Genomic data from new *S. pombe* isolates will reveal genetic features that are unique to these yeasts.

The collection of microorganisms of the Magarach Institute of Viticulture and Winemaking includes a large number of yeast and bacterial strains isolated from various winemaking regions. The *S. pombe* strain I-540 was isolated in 1940 from grapes in the Maykop region of the Northern Caucasus (Russia). This strain has an alcohol tolerance limit of 16% (vol/vol) and is capable of carrying out MAF at pH 3 and lower (5). The strain is promising for use in winemaking to reduce the acidity of wine (5).

Here, we report the draft genome of *S. pombe* I-540. The strain was grown in YPD medium at 28°C for 2 days. Genomic DNA was extracted using DNeasy PowerSoil DNA Isolation Kit (Qiagen, Germany). Sequencing of NEBNext Ultra II DNA Library (New England Biolabs, Ipswich, USA) was performed using Illumina MiSeq with MiSeq Reagent Kit v.3 (Illumina Inc., San Diego, USA) and produced 1,717,491 read pairs (2 × 300 nt). Adapter sequences were removed using Cutadapt v.1.8 (6), overlapping paired reads were merged using FLASH v.1.2.11 (7), and low quality read ends were trimmed by Sickle v.1.33 (8) using q = 30 quality threshold parameter. Illumina reads were assembled using SPAdes v.3.15.4 (9) with—isolate parameter. Gene structures were predicted by Augustus v.3.4.0 (10) with *S. pombe* trained model. BLAT v.34 (11) splice-aware alignments of *S. pombe* strain 972h− genes to the assembled contigs were incorporated to Augustus workflow as hints to improve intron-exon predictions. Genome completeness was assessed using BUSCO v.5.7.1 (7) with fungi database. Genome statistics are described in Table 1.

**TABLE 1:** Genome sequencing statistics

Parameter	Value
Illumina sequencing	
Reads number	3,434,982
Total bases (bp)	1,033,929,582
Genome assembly	
Total base	12,518,769 bp
Number of contigs	674
The longest contig	1,211,609 bp
Contig N50	330,986 bp
GC content (%)	36.06
Protein-coding genes	4,781
tRNA genes	168
Non-coding DNA (%)	45.01
Genome Coverage	50×
Copies of rRNA gene cluster	about 79
Genome assembly assessment	
Complete BUSCOs	94.5%
Fragmented BUSCOs	1.6%
Missing BUSCOs	3.9%

The genes for MAF have been identified, including the *MAE2* gene encoding a malic enzyme. This genomic data will be useful for comparative genomics studies of *S. pombe* and application of these yeasts in winemaking.

## Data Availability

This BioProject has been deposited in GenBank under accession number PRJNA1019646. The draft genome of nucleotide sequence of *Schizosaccharomyces pombe* strain I-540 has been deposited in DDBJ/ENA/GenBank under the accession number JBHHLB000000000, and raw reads have been deposited in the National Center for Biotechnology Information Sequence Read Archive under the accession number SRX21855654. The version described in this paper is the first version.
